# 

**Published:** 1809-08

**Authors:** 


### NEW MEDICAL PUBLICATIONS.

Reports on the Effects of a peculiar Regimen on Schirrous Tumors and Cancerous Ulcers. By W. Lambe, M. D. 8vo. 5s.

A System of Surgery. By James Russell, F. R. S. Edin. and Professor of Clinical Surgery in the University of Edinburgh, 4 vols. 8vo.

The Vaccine Scourge, No. II. 1s.

---

### CORRESPONDENCE.

We are very glad to find Mr. Ward purposes collecting his scattered remarks on the external use of opium into a pamphlet, which we are taught to hope may appear in the course of the summer. He is perfectly at liberty to avail himself of whatever Remarks our Journal may afford him in the prosecution of his valuable undertaking.

Our Lincolnshire Correspondent cannot expect us to insert his favour in its present form; we have, however, referred it to our Reviewer, who will avail himself of the hints contained in it when the work to which it relates comes before him.

We are much obliged to Mr. Scott for the many useful hints contained in his paper, p. 94, of this Number; but we must be allowed to insert anonymous communications of useful information, provided they do not reflect on any of those names which we are permitted to publish. Mr. Scott may be reminded:

"Scilicet est aliquid cum sævis vivere ventis;

"Ingenio populi convenit ipse sui." Ovid.

Though not a fighting man, he may acquire courage from the element in which he lives, and from the heroes who form his society. But the modest youth, who has scarcely emerged from College, and the lectures of his Professor, must be allowed to make his first efforts unknown, though he may hope not unobserved.

We wish very much to call the attention of the Medical Public to the contagious property of certain sore throats, if such is really the case. It is well known that Dr. Pitcairn was seized with the complaint which proved fatal to him about a week after he had been watching with peculiar attention a patient who died under an exactly similar complaint. Our readers will recollect Dr. Ferguson's case, with our remarks, and his polite notice of them. In the middle of the last month died Sir John Macnamara Hayes, of a sore throat, with symptoms in most respects similar to Dr. Pitcairn's, and with a similar appearance on dissection. We cannot learn that he was exposed to a similar disease. We shall be glad of any information concerning analogous cases.

